# Navigated versus conventionally instrumented total knee arthroplasty techniques: No difference in functional alignment or balance

**DOI:** 10.1002/ksa.12557

**Published:** 2024-12-06

**Authors:** Shane P. Russell, Sarah Keyes, Grant Grobler, James A. Harty

**Affiliations:** ^1^ Department of Orthopaedics Bon Secours Hospital Cork Cork Ireland; ^2^ Department of Orthopaedics Cork University Hospital Cork Ireland; ^3^ Royal College of Surgeons in Ireland Dublin Ireland; ^4^ University College Cork Cork Ireland

**Keywords:** conventional instrumentation, inverse kinematic alignment, kinematic alignment, navigated total knee arthroplasty, personalised arthroplasty, total knee arthroplasty

## Abstract

**Purpose:**

Much debate exists about the superiority of navigated versus conventional instrumentation for achieving optimal balance and alignment during total knee arthroplasty (TKA). Recent registry data indicate no long‐term survivorship benefit for TKAs performed using technology assistance, despite the added resource and financial costs. However, outcome comparisons are confounded by varying surgeon techniques and targets for ideal balance and alignment. This study aimed to investigate alignment or balance outcome differences between navigated and conventionally instrumented TKAs performed using an identical operative sequence and alignment strategy.

**Methods:**

Fifty navigated and 50 conventionally instrumented primary TKAs, using an identical inverse kinematic alignment strategy, were included. Navigation equipment was used intraoperatively to ‘post‐cut’ record the conventionally instrumented TKAs. Intraoperative balance, range, and alignment; and post‐operative radiographic accuracy for restoration of constitutional alignment were compared.

**Results:**

Forty‐nine navigated and 49 conventionally instrumented TKAs were compared (*n* = 2 excluded due to inadequate radiographs). No preoperative demographic or deformity severity differences existed. No intraoperative balance, range or alignment difference existed. Neither technique was more accurate for restoration of constitutional alignment.

**Conclusion:**

Whilst large registry data may be confounded by uncaptured variables such as surgeon balancing techniques or surgeon alignment strategy preferences, this study found no alignment or balance differences between navigated versus conventionally instrumented TKA techniques for a surgeon and technique‐controlled study. Although the increased resources necessary for technology assistance are not justified by this study, further studies may identify significance using larger samples or comparison of alternative outcomes.

**Level of Evidence:**

Level II.

**Abbreviations**
aHKA: arithmetic hip–knee–ankle
AI: artificial intelligence
CPAK: coronal plane alignment of the knee
FFD: fixed flexion deformity
HKA: hip–knee–ankle
iKA: inverse kinematic alignment
JLCA: joint line convergence angle
JLO: joint line obliquity
MAD: mechanical axis deviation
mLDFA: mechanical lateral distal femoral angle
MPTA: medial proximal tibial angle
NS: not significant
SD: standard deviation
TKA: total knee arthroplasty
UK: United Kingdom
USA: United States of America
WA: Washington

## INTRODUCTION

Much debate exists about the superiority of computer‐assisted surgery, such as navigated or robotic instrumentation, versus conventional instrumentation for achieving optimal balance and alignment during total knee arthroplasty (TKA) [4, 9, 12, 16, 17, 20, 21, 23, 28].

Recent registry data indicate no long‐term implant survivorship benefit for TKAs performed using technology assistance, despite the added resource and financial costs [16, 22, 27]. However, large registry outcome comparisons are confounded by variables left uncaptured by those registries, such as: surgeon skill and experience, preoperative anatomical and disease‐related variations; surgical technique variations and varying surgeon philosophies for ideal balance and alignment during TKA. For example, while the Australian Orthopaedic Association National Joint Replacement Registry comparisons adjust for ‘age, gender, American Society of Anesthesiologists, body mass index, bearing surface, patella component usage and stability’ when comparing navigated versus conventionally instrumented TKAs, it cannot adjust for uncaptured surgeon variables such as preferred gap targets, or mechanical versus personalised alignment strategies [27]. Similarly, disadvantages of large multi‐centre, multi‐surgeon randomised controlled trials include an inability to control for such confounding inter‐surgeon variables [9].

Accuracy of implant positioning remains crucial, among all alignment strategies, for long‐term implant survival [7, 8]. Additionally, improved knee function, reduced pain scores and improved quality of life are consistently correlated with the accuracy of achieving the target alignment and balance [6, 7, 8].

This study aimed to investigate whether alignment or balance differences exist between navigated and conventionally instrumented TKAs performed using an identical, surgeon and centre‐controlled operative sequence and alignment strategy. It was therefore hypothesized that no alignment or balance differences exist for an identical TKA strategy performed using either navigation or conventional instrumentation.

## METHODS

All patients undergoing TKA for primary, end‐stage osteoarthritis at a single centre by a single surgeon were included for alternate allocation to undergo either: (1) Navigated TKA or (2) Conventionally instrumented TKA with navigation equipment recording. Bilateral, complex primary, and revision procedures were excluded, as were TKAs indicated by conditions other than end‐stage osteoarthritis. A non‐consecutive series is represented as a maximum of two patients per operating list were enrolled.

### Surgical technique

The *Attune Knee System* (DePuy Orthopaedics) and *Brainlab Knee 3* (Smith & Nephew) image‐free navigation setup was used for all cases (both navigated and conventionally instrumented procedures).

The same operative sequence and inverse kinematic alignment (iKA) technique were used for both groups, which included patellar resurfacing in all cases. The medial proximal tibial angle (MPTA) was restricted to 84–92° in keeping with previously published boundaries, which have shown no adverse effects on implant survivorship and represent 93% of native Caucasian MPTAs [1, 11, 13, 25, 29, 30].

Independent of allocation to either the navigated or conventionally instrumented groups, patients received according to surgeon preference, either: (1) if bone quality allowed, cementless, rotating platform, cruciate retaining implants, or (2) in cases of poor bone quality, cemented, fixed bearing, posterior stabilised implants.

*Navigated TKAs* were performed using iKA methods with all bone cuts performed using computer navigation for planning. After standard setup and medial parapatellar approach, a conservative distal femoral cut was performed, followed by a definitive tibial resection. A definitive distal femoral resection was subsequently performed to achieve extension gap balance. Posterior femoral cuts were performed to achieve satisfactory flexion gap balance before femoral chamfer, box, tibial keel and patellar preparations. Implants were trialled, and components were implanted with final case navigation data stored for analysis.

*Conventionally instrumented TKAs with navigation recording* were performed using the same iKA alignment strategy as the navigated knees. For this cohort, although the navigation equipment was set up identically to the navigated TKAs, all bone cuts were planned and executed according to the conventional instrumentation jigs, as the authors have detailed in a previous report [24]. The navigation equipment was employed for ‘post‐cut recording’ only and never for ‘pre‐cut guidance’. The iKA operative sequence was identical to that described for the navigated group, with bony cuts this time performed using intramedullary femoral jigs and extramedullary tibial jigs. Immediately after each jig‐guided femoral or tibial cut, the navigation equipment was used to document the position of those conventional cuts. The procedure thus proceeded as a routine conventionally instrumented TKA without the influence of the navigation readings on intraoperative decision‐making.

For both techniques, recorded navigation data included: patient demographics, implant details, final gap sizes at maximum extension and at 90° flexion; coronal tibial component position (as a proxy for the MPTA); final hip–knee–ankle (HKA); and preoperative and final knee maximum extension.

All patients received preoperative and 6‐week post‐operative bilateral long leg radiographs using the ‘stand to attention’ position with patellae facing forward. Radiographic analysis was performed using a validated artificial intelligence (AI) method to calculate anatomic measurements of interest (96% reproducibility) (ImageBiopsy Lab), including the HKA, mechanical lateral distal femoral angle (mLDFA), MPTA and the joint line convergence angle (JLCA) (Appendix A) [15, 26].

Comparison was made between groups for: (1) preoperative differences in demographics and deformity severity; (2) intraoperative difference in balance, alignment and range of motion and (3) post‐operative restoration of constitutional alignment.

To compare the accuracy of techniques for restoring prearthritic alignment, two methods were used to estimate the patient's constitutional coronal alignment. First, the arithmetic HKA (aHKA) was calculated for use as an accurate predictor of constitutional HKA according to MacDessi et al.'s description (the mLDFA is subtracted from the MPTA thereby predicting the HKA in the presence of a normal JLCA) [18]. Comparison was then made between the preoperative aHKA (target alignment) and the post‐operative HKA (achieved alignment).

Second, the coronal alignment of the nonoperative leg was used as an approximate target for constitutional alignment of the operative limb (although cognisant of a normal population HKA symmetry rate of 79%) [2]. Post‐operative radiographs were examined to exclude nonoperative knees with implants, abnormally widened JLCAs indicative of joint space narrowing due to wear or ligamentous incompetency (0.51 ± 1.05° accepted as normal), or evidence of previous realignment osteotomies [3].

*Microsoft Excel* was used for data storage, descriptive statistical analysis, *t* test for parametric data, Mann–Whitney *U* test for non‐parametric data, *F* test and Levene's test for variance; with *p* > 0.05 treated as not significant (NS).

## RESULTS

One hundred patients were enrolled (50 navigated and 50 instrumented). Two patients were excluded due to inadequate radiographs for analysis. Ninety‐eight patients were therefore included for analysis, representing 49 navigated and 49 conventionally instrumented procedures.

### Preoperative comparisons

There were no significant preoperative differences between cohorts for age, gender or side (Table 1).

**Table 1:** Preoperative group comparison, standard deviation (SD).

	Demographics			Preoperative deformity indicators			
	Age (years) x̅ (±SD)	Sex	Side	FFD (°) x̅ (±SD)	JLCA (°) x̅ (±SD)	MAD (mm) x̅ (±SD)	HKA (°) x̅ (±SD)
Navigated	67.24 (±9.23)	26 Male 23 Female	23 Left 26 Right	5.14 (±5.19)	2.10 (±2.73)	9.04 (±21.02)	2.67 (±6.33)
Conventional	67.02 (±9.88)	21 Male 28 Female	28 Left 21 Right	5.05 (±4.22)	2.02 (±3.45)	8.93 (±19.82)	2.79 (±5.88)
Significance	NS (*p* = 0.45)	NS (*p* = 0.19)	NS (*p* = 0.19)	NS (*p* = 0.46)	NS (*p* = 0.45)	NS (*p* = 0.49)	NS (*p* = 0.47)

Abbreviations: FFD, fixed flexion deformity; HKA, hip–knee–ankle; JLCA, joint line convergence angle; MAD, mechanical axis deviation; NS, not significant.

**Figure 1:**
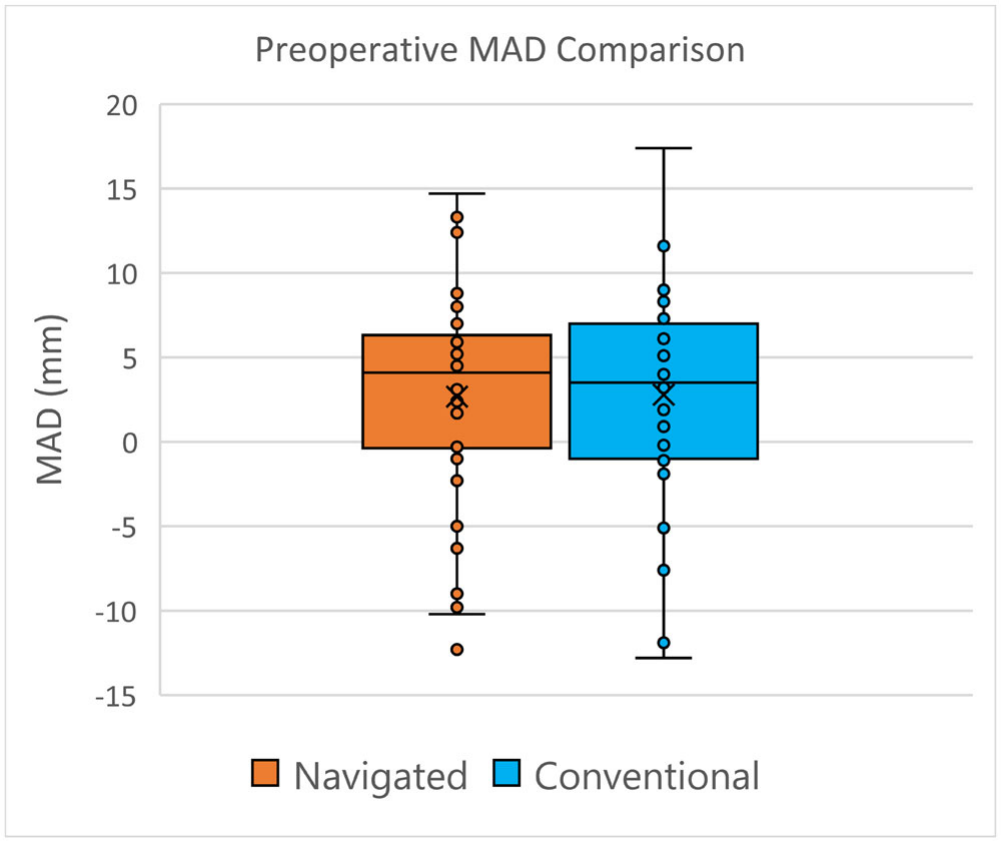
Preoperative MAD comparison. MAD, mechanical axis deviation.

For indicators of preoperative deformity severity, there was no difference between groups for fixed flexion deformity (FFD) nor for JLCA widening. The mechanical axis deviation (MAD) was similarly distributed between groups (Figure 1, Table 1).

**Figure 2:**
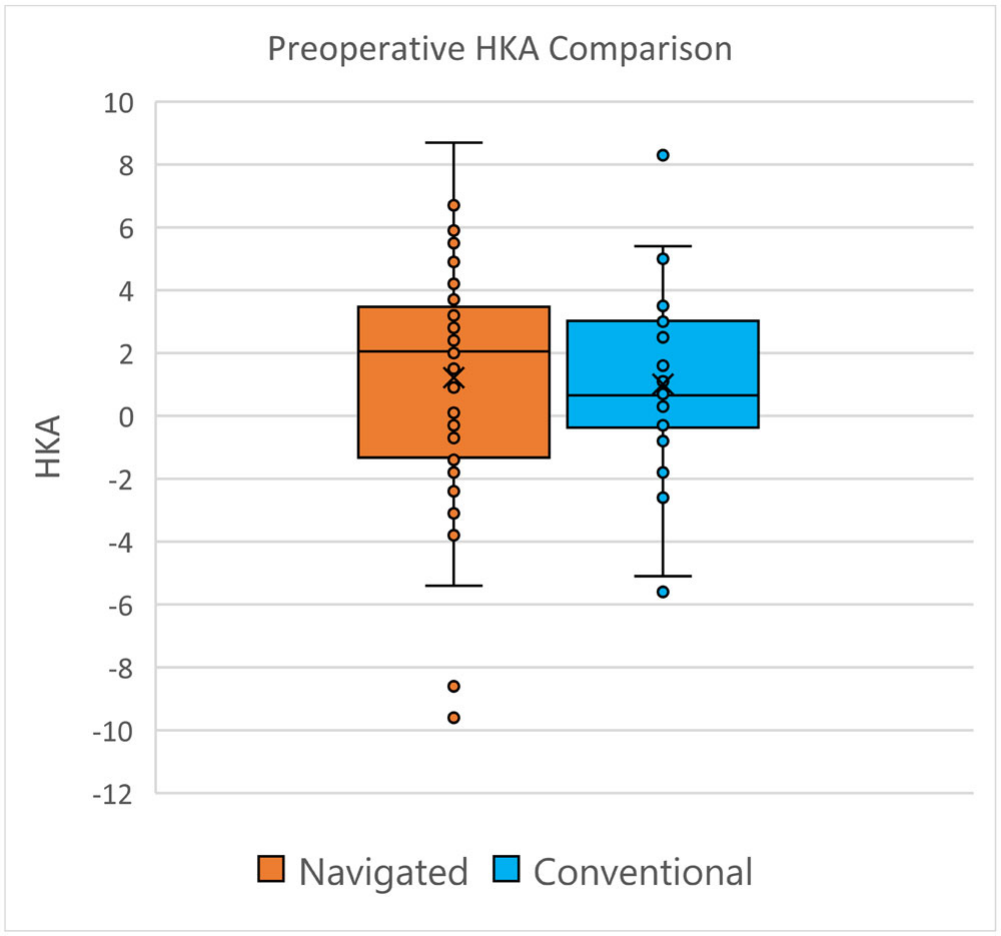
Preoperative HKA comparison. HKA, hip–knee–ankle.

The classically described coronal lower limb alignment profiles (valgus, neutral and varus) were similarly distributed (Figure 2). Two valgus outliers existed within the navigated group, with one varus and one valgus outlier within the conventional group (Figure 2).

**Figure 3:**
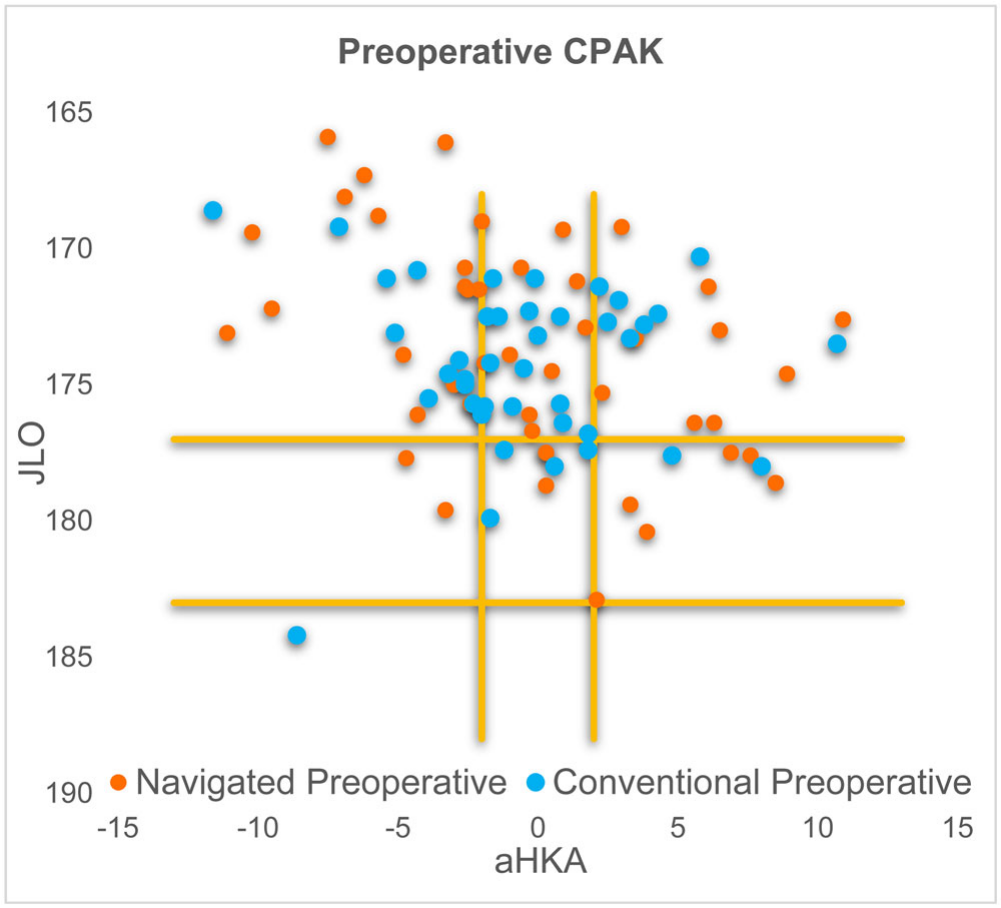
Preoperative CPAK. aHKA, arithmetic hip–knee–ankle; CPAK, coronal plane alignment of the knee; JLO, joint line obliquity.

Preoperative lower limb alignment profiles were similarly distributed according to the coronal plane alignment of the knee (CPAK) classification (Figure 3) [19].

### Intraoperative comparisons

**Figure 4:**
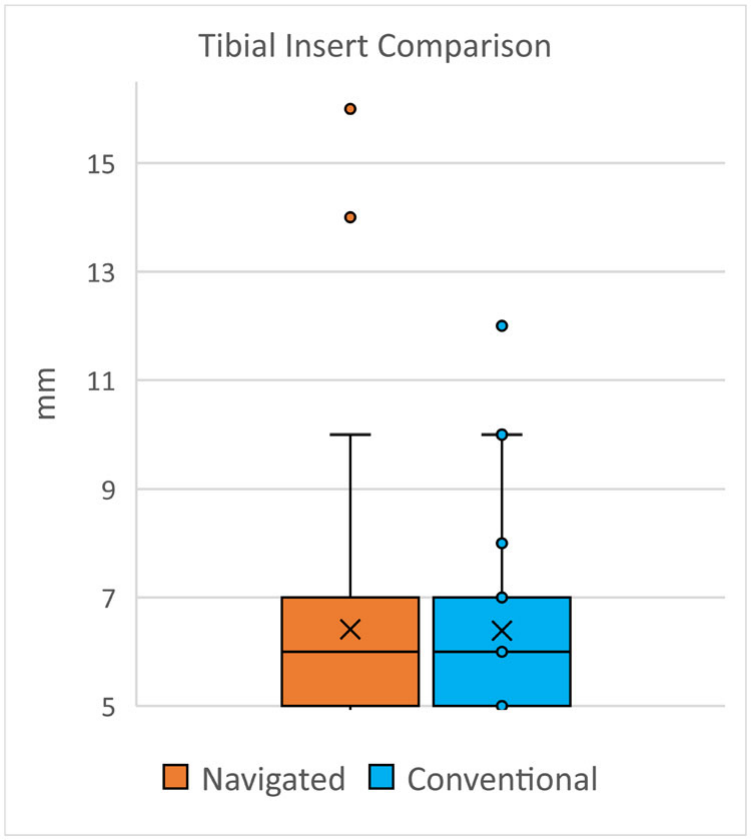
Tibial insert height.

As a proxy for tibial bone resection volume, there was no tibial insert height difference between groups (mean navigated: 6.43 [±2.56] mm; conventional: 6.39 [±1.50] mm; NS [*p* = 0.46]) (Figure 4).

**Figure 5:**
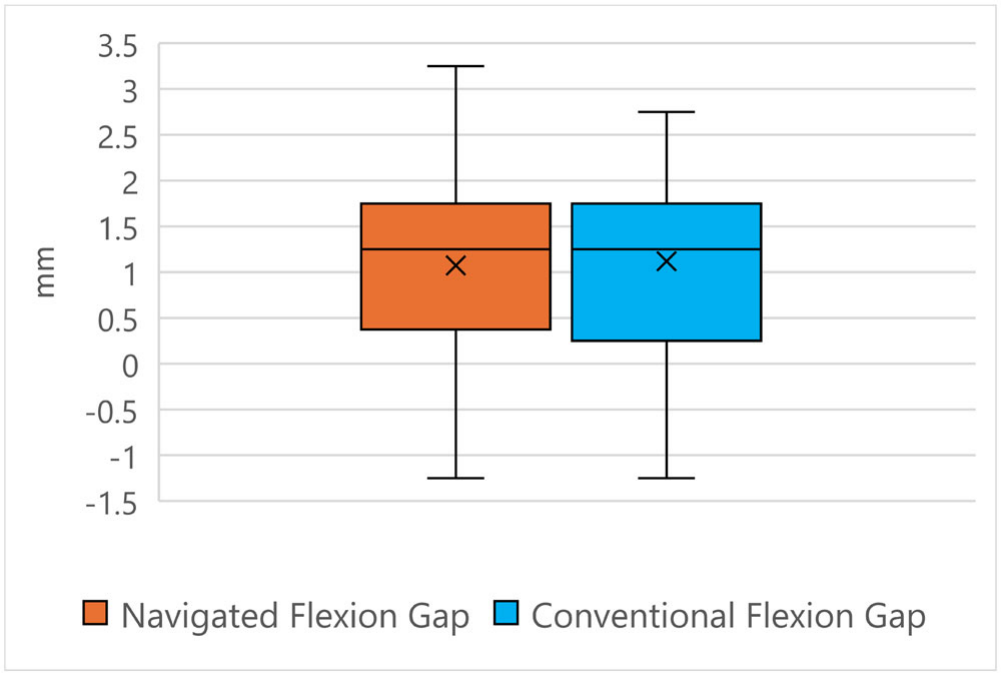
Flexion gap comparison.

**Figure 6:**
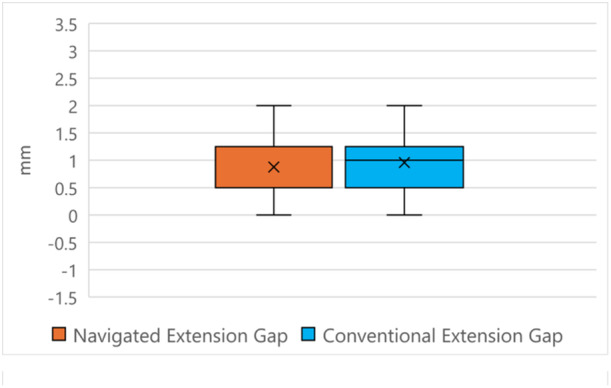
Extension gap comparisons.

**Figure 7:**
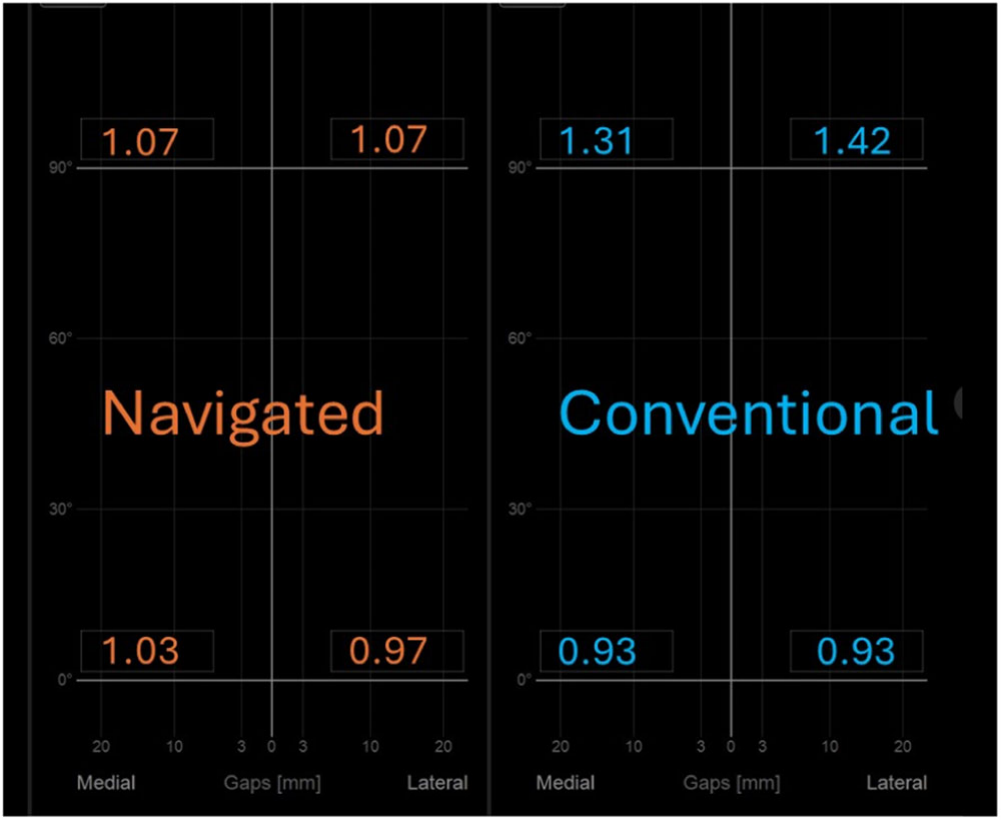
Mean gap values for navigated and conventionally instrumented TKAs (no significant differences). TKA, total knee arthroplasty.

Comparing intraoperative gap measurements, there was no difference between navigated or conventionally instrumented knees for mean gap values among the four‐gap quadrants (Table 2, Figures 5, 6, 7). For mediolaterally combined values, there were no differences between mean flexion or extension gaps (Figures 5 and 6). However, there was a trend for conventionally instrumented knees to return tighter extension gaps (0.93 [±1.25] mm vs. 1.00 [±1.40] mm, NS [*p* = 0.32]) and looser posterolateral flexion gaps (1.07 [±1.18] mm vs. 1.42 [±1.37] mm, NS [*p* = 0.10]) (Tables 2 and 3). There was no difference in flexion or extension imbalances between the two cohorts (Table 4). Both techniques yielded similar flexion–extension gap mismatch values (Table 5).

**Table 2:** Comparison of mean quadrant gap values.

	Lateral flexion gap (mm) x̅ (±SD)	Medial flexion gap (mm) x̅ (±SD)	Lateral extension gap (mm) x̅ (±SD)	Medial extension gap (mm) x̅ (±SD)
Navigated	1.07 (±1.18)	1.07 (±1.32)	0.97 (±1.61)	1.03 (±1.43)
Conventional	1.42 (±1.37)	1.31 (±1.23)	0.93 (±1.50)	0.93 (±1.29)
Significance	NS (*p* = 0.10)	NS (*p* = 0.18)	NS (*p* = 0.45)	NS (*p* = 0.36)

Abbreviations: NS, not significant; SD, standard deviation.

**Table 3:** Comparison of mediolaterally combined gap values.

	Flexion gap (mm) x̅ (±SD)	Extension gap (mm) x̅ (±SD)
Navigated	1.07 (±1.12)	1.00 (±1.40)
Conventional	1.36 (±1.12)	0.93 (±1.25)
Significance	NS (*p* = 0.10)	NS (*p* = 0.39)

Abbreviations: NS, not significant; SD, standard deviation.

**Table 4:** Comparison of flexion and extension gap imbalances.

	Mean flexion imbalance (mm) x̅ (±SD)	Mean extension imbalance (mm) x̅ (±SD)
Navigated	0.90 (±0.68)	0.88 (±0.87)
Conventional	1.04 (±0.81)	0.96 (±0.80)
Significance	NS (*p* = 0.17)	NS (*p* = 0.32)

Abbreviations: NS, not significant; SD, standard deviation.

**Table 5:** Mean flexion–extension mismatch.

	Mean flexion–extension mismatch (mm) x̅ (±SD)
Navigated	1.04 (±0.88)
Conventional	1.28 (±0.94)
Significance	NS (*p* = 0.10)

Abbreviations: NS, not significant; SD, standard deviation.

**Figure 8:**
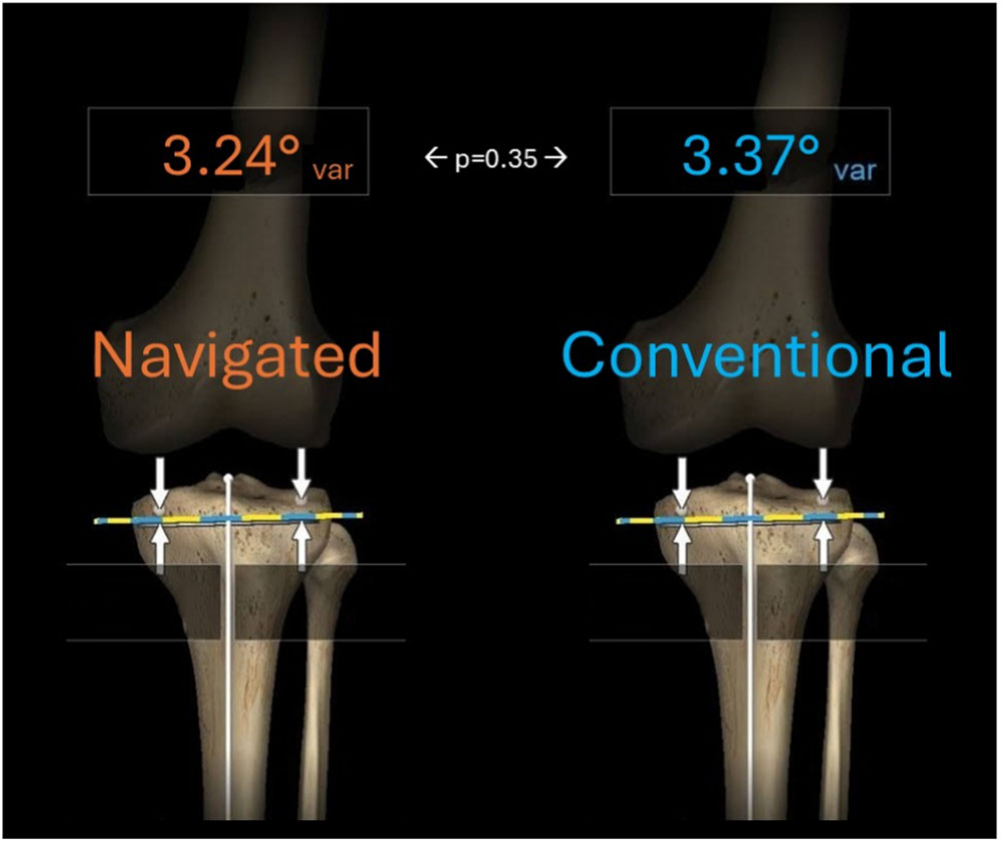
Tibial component coronal plane: Representation of intraoperative mean values.

**Figure 9:**
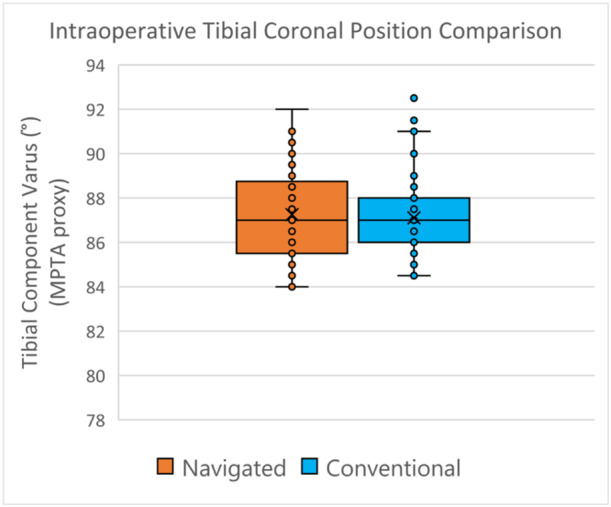
Distributions of tibial component coronal plane intraoperative verifications.

With regard to tibial component positioning, as measured intraoperatively, there was no difference in the mean or variance of tibial coronal position between navigated or conventionally instrumented knees (3.24 [±1.64]° vs. 3.37 [±1.34]° respectively, NS [*p* = 0.35]) (Figures 8 and 9). Six per cent (*n* = 3) of navigated knees and 2% (*n* = 1) of conventional knees exceeded the tibial component varus restriction (MPTA < 85°), while no TKA exceeded the tibial component valgus restriction (MPTA > 92°).

### Post‐operative comparison

The mean difference between the intraoperatively measured MPTA using the navigation system, for both groups, and the post‐operatively measured MPTA using AI radiographic measurements was 1.49 ± 1.18° (intraoperative: 87.18 ± 1.92°, post‐operative: 88.24 ± 2.31°; significant with *p* < 0.01).

Compared to preoperative measurements, navigated and conventionally instrumented procedures resulted in similar interval alignment changes (Table 6).

**Table 6:** Comparison of preoperative to post‐operative deformity indicators changes.

	ΔHKA (°) x̅ (±SD)	ΔMAD (mm) x̅ (±SD)	ΔLDFA (°) x̅ (±SD)	ΔMPTA (°) x̅ (±SD)	ΔJLO (°) x̅ (±SD)	ΔJLCA (°) x̅ (±SD)	ΔFFD (°) x̅ (±SD)
Navigated	2.76 (±3.32)	8.70 (±11.13)	1.15 (±2.37)	2.25ׄ (±2.66)	2.77 (±3.55)	2.42 (±1.55)	2.92 (±5.57)
Conventional	2.41 (±3.87)	7.51 (±12.36)	0.40 (±2.23)	2.23 (±2.21)	1.90 (±2.94)	3.01 (±1.87)	2.49 (±4.33)
Significance	NS (*p* = 0.33)	NS (*p* = 0.32)	NS (*p* = 0.07)	NS (*p* = 0.49)	NS (*p* = 0.11)	NS (*p* = 0.06)	NS (*p* = 0.34)

Abbreviations: FFD, fixed flexion deformity; HKA, hip–knee–ankle; JLCA, joint line convergence angle; JLO, joint line obliquity; LDFA, lateral distal femoral angle; MAD, mechanical axis deviation; MPTA, medial proximal tibial angle; NS, not significant; SD, standard deviation.

There was no difference between groups for improvements between preoperative and post‐operative maximum knee extension (Table 6).

**Figure 10:**
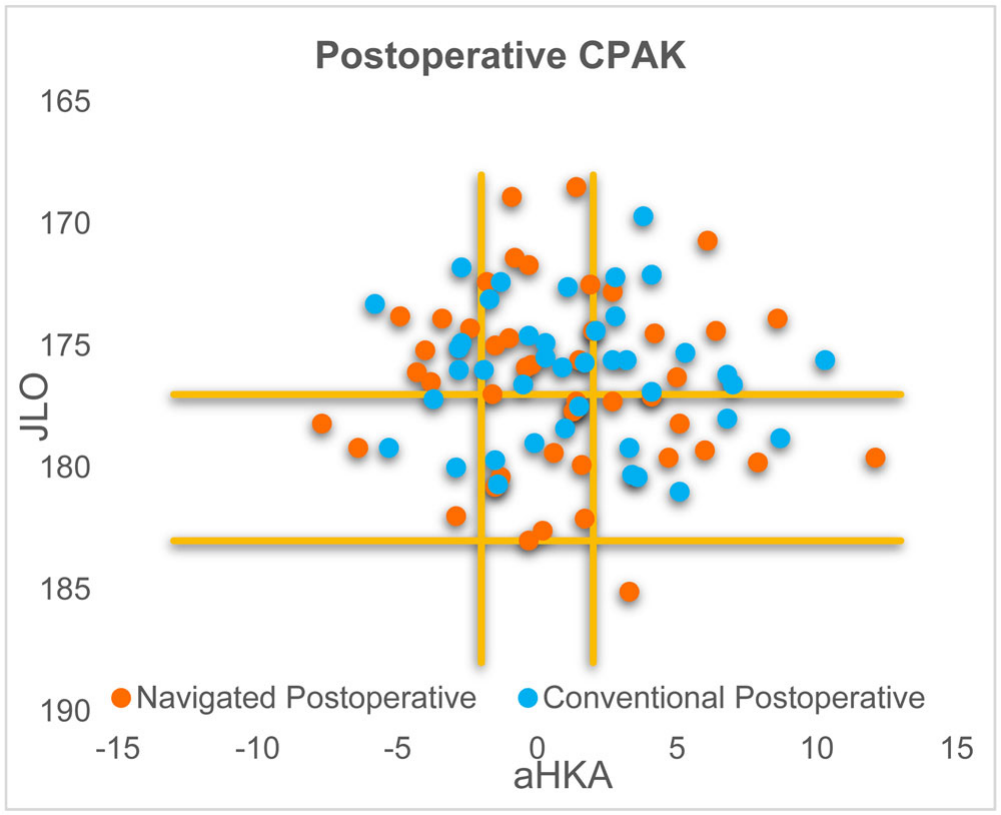
Post‐operative CPAK.

Post‐operative CPAK profiles were similarly distributed between the navigated and conventional groups (Figure 10).

Examining the superiority of navigated versus conventional instrumentation for restoring constitutional alignment by employing the aHKA as the target HKA, there was no difference between methods for target accuracy. Although conventional knees returned HKAs closer to constitutional profiles, this was not significant (Table 7).

**Table 7:** Accuracy of navigated versus conventional techniques for achieving constitutional alignment. Inaccuracy: Average difference between target and achieved HKA.

	Preoperative HKA (°) x̅ (±SD)	Target (aHKA) (°) x̅ (±SD)	Post‐operative HKA (°) x̅ (±SD)	Inaccuracy (°) x̅ (±SD)
Navigated	2.67 (±6.33)	0.57 (±4.48)	0.01 (±3.83)	2.58 (±1.90)
Conventional	2.79 (±5.88)	0.77 (±3.72)	−0.47 (±3.33)	2.33 (±2.22)
Significance	NS (*p* = 0.47)	NS (*p* = 0.41)	NS (*p* = 0.27)	NS (*p* = 0.29)

Abbreviations: aHKA, arithmetic hip–knee–ankle; HKA, hip–knee–ankle; NS, not significant; SD, standard deviation.

Secondarily, using the normal contralateral limb as an approximate control for constitutional alignment after exclusions (knee implants: *n* = 13, abnormal JLCA: *n* = 51), there was no difference between the efficacy of navigated or conventional instrumentation for restoring the constitutional HKA (NS, *p* = 0.27).

## DISCUSSION

This study found no difference in functional alignment or balance outcomes between navigated and conventionally instrumented TKAs performed using an identical operative strategy.

First, a preoperative difference, or selection bias, between cohorts was not identified. No differences were detected between groups for: (1) patient factors such as age, sex or sidedness; nor for (2) radiographic indicators of disease severity such as HKA, MAD or JLCA distributions; nor for (3) preoperative FFD.

Regarding intraoperative comparisons, no difference between techniques was found for: absolute gap values; flexion gap balance; extension gap balance nor flexion–extension gap mismatches.

There was no difference in polyethylene insert thickness between groups, indicating a similar volume of tibial bone was resected using either technique. This study's correlation between insert thickness and tibial bone resection assumes an accurate joint line restoration in a well‐balanced knee. As an identical iKA technique was employed for both groups, and all knees were well‐balanced, the authors find this proxy for tibial bone resection volume to be well‐founded and may be useful for future studies.

When examining accuracy for achieving the target constitutional alignment, neither technique was found to be more accurate when using either the validated aHKA method or, less reliably, the contralateral limb as a control. Additionally, there was no difference between groups for interval changes to the HKA, MAD, LDFA, MPTA or JLCA. That is, navigated or conventionally instrumented TKAs resulted in the same coronal lower limb alignment profile changes required for restoration of constitutional alignment in the presence of preoperative deformity. The relative preservation of the LDFA compared to the MPTA in both groups is consistent with a successful kinematic restorative strategy [15].

This study has several limitations which first include a single‐centre, single surgeon design. However, in keeping with the aims of this study, it was of critical importance to reduce the confounding variables of inter‐surgeon or inter‐departmental variations. An identical iKA technique for both groups was also employed, rather than accepting confounding variations of technique between navigated or conventional methods. This study's reliability and reproducibility of results are improved by control of such potentially confounding variables.

Second, this study may have been underpowered, with no differences found between groups for all observed comparators. This is the first study utilising the described methodology of navigation recording during conventional instrumentation for direct comparison of techniques. As such, a power analysis was rejected as insufficient analogous or applicable prior research was available to form a reliable estimate of effect size and variance. Further studies may identify significance by employing the findings of the present study for power analyses whilst observing larger population samples.

Third, the authors recognise the low rate (79%) of HKA symmetry in a normal population sample [2]. However, this secondary analysis was performed after noting no difference existed between groups using the validated aHKA method, and the authors would not advocate for the use of this method alone.

**Figure A1:**
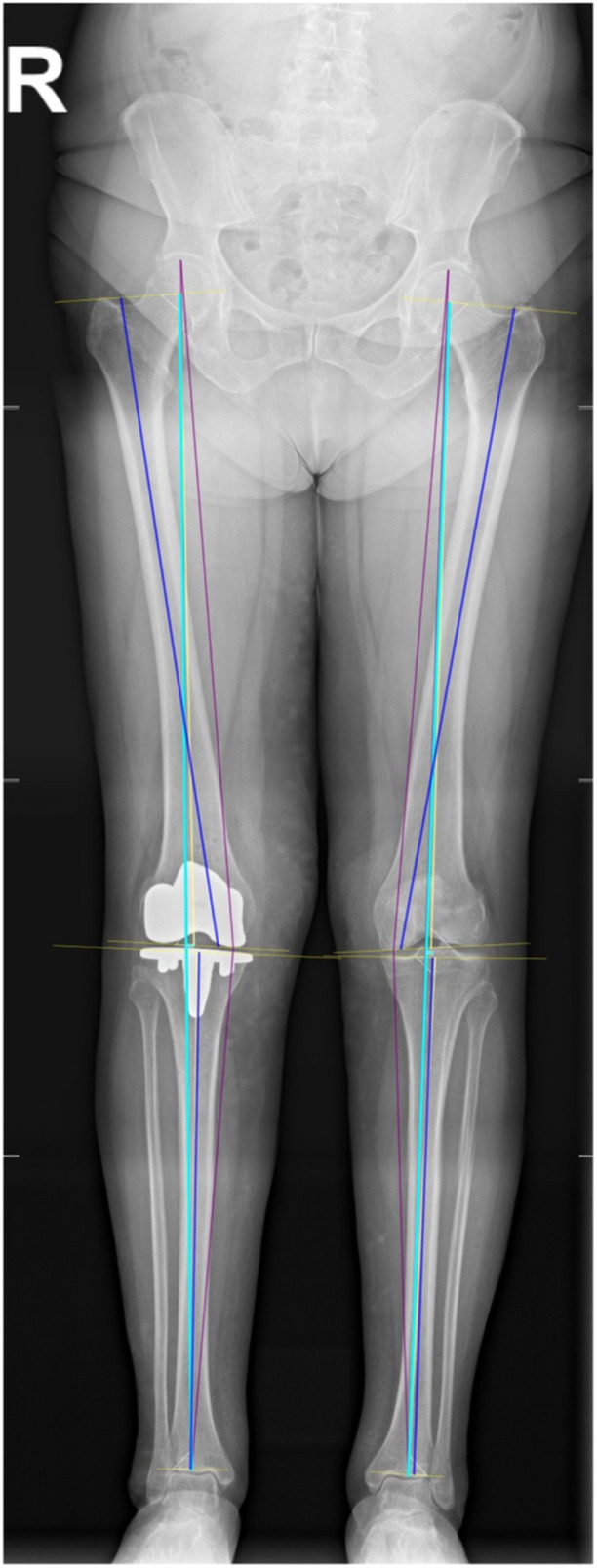
Example radiographic analysis.

Causes for a 1.49° mean difference between the intraoperative navigation MPTA and the post‐operatively radiographic MPTA may include the inaccuracies of both systems and that of AI analysis (96% reproducible) (Figure A1) [26]. The radiographically measured MPTA has been demonstrated to be within 0.8° with ±10° leg rotation [14, 26]. In addition, patients lacking full post‐operative knee extension may introduce radiographic measurement inaccuracies [5]. Previous studies comparing intraoperative navigation MPTA measurements to manual post‐operative radiographic measurements have identified larger discrepancies [10].

Fink et al. describe similar findings during a surgeon and alignment strategy‐controlled comparison of robotic versus conventional instrumentation for the accuracy of restoration of constitutional alignment [21]. Turan et al. describe for a retrospective TKA cohort comparison, robotic instrumentation yielded post‐operative radiographic measurements that were favourable to matched conventionally instrumented TKAs [28]. However, for that restricted kinematic alignment technique, no comparison to the target constitutional alignment is made, which raises the question of how alignment superiority should be defined. This study, in contrast, utilised both the validated and accurate aHKA method for constitutional alignment control, in addition to a secondary comparison to the normal contralateral limb, where no superiority of techniques was identified.

To the authors' knowledge, this is the first reported study to use intraoperative navigation recording of conventionally instrumented TKAs for any alignment strategy comparison. Future studies may wish to use this method.

## CONCLUSION

Whilst large registry data may be confounded by uncaptured, confounding variables such as surgeon alignment strategy preferences or surgeon balancing techniques, this study found no alignment or balance differences between navigated or conventionally instrumented TKA techniques for a surgeon, technique and alignment strategy‐controlled study. Surgeons may be reassured that similar outcomes may be achieved by utilising either technique. Although the increased resources necessary for technology assistance are not justified by this study, further studies may identify significance using larger samples or comparison of alternative outcomes such as functional scores, patient‐reported outcomes or long‐term survivorship [12, 22].

## AUTHOR CONTRIBUTIONS

**Shane P. Russell:** Investigation; methodology; material preparation; data collection; data analysis; writing. **Sarah Keyes:** Investigation; material preparation; data collection; writing. **Grant Gobler:** Investigation; data collection. **James A. Harty:** Conceptualization; resources; investigation; methodology; data collection; supervision.

## CONFLICT OF INTEREST STATEMENT

The authors declare no conflicts of interest.

## ETHICS STATEMENT

Ethical approval was not sought for this audit of practice.

## Data Availability

Research data are not shared.

## APPENDIX A

See Figure A1.
